# Using implementation science to develop and implement a guideline to reduce unnecessary preoperative testing for asymptomatic bacteriuria prior to elective arthroplasty

**DOI:** 10.5194/jbji-6-57-2020

**Published:** 2020-12-21

**Authors:** Judith S. L. Partridge, Madeleine Daly, Carolyn Hemsley, Zameer Shah, Krishanthi Sathanandan, Cathryn Mainwaring, Jugdeep K. Dhesi

**Affiliations:** 1 Department of Ageing and Health, Guy's and St Thomas' NHS Foundation Trust, London, UK; 2 Division of Primary Care and Public Health Sciences, Faculty of Life Sciences and Medicine, King's College London, London, UK; 3 Department of Infection Diseases, Guy's and St Thomas' NHS Foundation Trust, London, UK; 4 Department of Trauma and Orthopaedics, Guy's and St Thomas' NHS Foundation Trust, London, UK; 5 Whipps Cross Hospital, Barts Health NHS Trust, London, UK; 6 Department of Geriatric Medicine, King's College Hospital NHS Foundation Trust, London, UK; 7 Division of Surgery and Interventional Science, University College London, London, UK

## Abstract

**Introduction**: Guidelines and consensus statements do not support routine preoperative testing for asymptomatic bacteriuria (ASB) prior to elective arthroplasty. Despite this, urine testing remains commonplace in orthopaedic practice. This mixed methods stepwise quality improvement project aimed to develop and implement a guideline to reduce unnecessary preoperative testing for asymptomatic bacteriuria prior to elective arthroplasty within a single centre. **Methods**: Step 1 – description of current practice in preoperative urine testing prior to arthroplasty within a single centre; Step 2 – examination of the association between preoperative urine culture and pathogens causing prosthetic joint infection (PJI); Step 3 – co-design of a guideline to reduce unnecessary preoperative testing for asymptomatic bacteriuria prior to elective arthroplasty; Step 4 – implementation of a sustainable guideline to reduce unnecessary preoperative testing for asymptomatic bacteriuria prior to elective arthroplasty. **Results**: Retrospective chart review showed inconsistency in mid-stream urine (MSU) testing prior to elective arthroplasty (49 % preoperative MSU sent) and in antimicrobial prescribing for urinary tract infection (UTI) and ASB. No association was observed between organisms isolated from urine and joint aspirate in confirmed cases of PJI. Co-design of a guideline and decision support tool supported through an implementation strategy resulted in rapid uptake and adherence. Sustainability was demonstrated at 6 months. **Conclusion**: In this stepwise study, implementation science methodology was used to challenge outdated clinical practice, achieving a sustained reduction in unnecessary preoperative urine testing for ASB prior to elective arthroplasty.

**Figure 1:**
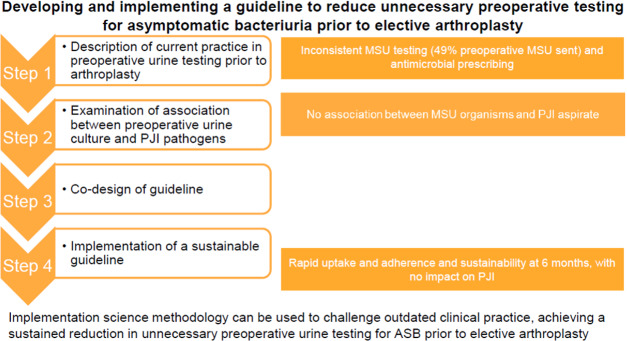
Graphic abstract.

## 1 Introduction

Arthroplasty is a common procedure, with over 250 000 performed in the UK in 2018 (NJR, 2019). Prosthetic joint infection (PJI) is a relatively uncommon but serious complication of arthroplasty, occurring in 1 %–2 % of cases (Izakovicova, 2019). It is associated with a 5-fold increase in 1-year mortality, deterioration in functional status, negative impact on quality of life, and significant financial cost (Izakovicova, 2019; Li, 2018). Such costs arise due to extended course antimicrobial treatment and the need for further surgical procedures (Nicolle, 2005; Middleton, 2019). Whilst urinary tract infection (UTI) is a well-described risk factor for PJI (Parvizi, 2019), the association between asymptomatic bacteriuria (ASB) and PJI is less well established.

ASB is defined as the presence of over 10${}^{5}$ colony-forming units per millilitre of bacteria in the urine without symptoms or signs of urinary tract infection (Nicolle, 2005). In the arthroplasty population, where many patients are older, the prevalence of ASB ranges from 15 % to 50 % (Nicolle, 2005). There is no evidence that treatment of ASB reduces rates of delirium, morbidity, or mortality (Parvizi, 2019; Abrutyn, 1994). Although a 3-fold increase in PJI has been reported in patients with ASB, no association is observed between pathogens cultured in the urine and those isolated from the infected arthroplasty. ASB is therefore unlikely to be a direct cause of PJI but rather a marker of susceptibility to infection in a vulnerable older population (Sousa, 2014; Weale, 2019). Furthermore, antimicrobial treatment for ASB does not affect rates of PJI (Sathanandan, 2019), and unnecessary antimicrobial treatment is associated with adverse outcomes, including C. *lostridioides difficile* infection and colonisation with multidrug-resistant organisms (Scott Israel, 2012; Cai, 2017). Such side effects are relevant in the often older arthroplasty population, with a number needed to harm from antibiotic treatment of just three in those aged over 65 years receiving antimicrobials for ASB (NICE, 2016; SIGN, 2012).

Guidance for preoperative urine screening is conflicting: National Institute of Clinical Excellence (NICE) preoperative guidelines recommend that urine testing, using a “dipstick”, should not form part of routine preoperative assessment (NICE, 2016), in keeping with the Scottish Intercollegiate Guidelines Network (SIGN), which does not recommend use of dipsticks in those aged over 65 years (SIGN, 2012). International consensus statements advocate cessation of routine urine testing in preoperative assessment prior to arthroplasty in the absence of symptoms of UTI (Parvizi, 2013). However, the British Orthopaedic Association continues to recommend routine urine testing prior to hip arthroplasty, irrespective of symptoms (BOAS, 2012). Although frequently used, urine dipsticks have a low sensitivity in the elderly population (Ninan, 2014).

As a result of this contradictory guidance, variation exists in the preoperative testing and management of ASB, resulting in delays to surgery, inconsistent antimicrobial prescribing, complications of antimicrobial use, and resultant cost (Mayne, 2016; David, 2000). Two-thirds of UK orthopaedic surgeons advocate preoperative treatment of ASB prior to arthroplasty, although 70 % were unable to cite evidence for this intervention (Finnigan, 2018).

Addressing the gap between evidence and practice requires an implementation science approach. Implementation science is the study of methods used to promote the adoption and integration of evidence-based practices, interventions and policies into routine healthcare (Nilsen, 2015; Forgarty International Center, 2020). Implementation science uses mixed methods to address key issues, including preparedness for change, capacity for change, stakeholder buy-in, strategy and sustainability (Palinkas, 2010; Braithwaite, 2014). Such an approach was used to inform this mixed-methods stepwise quality improvement project to develop and implement a guideline to reduce unnecessary preoperative testing for asymptomatic bacteriuria prior to elective arthroplasty.

### Objectives

- Step 1 – to describe current practice in preoperative urine testing prior to arthroplasty within a single centre
- Step 2 – to examine the association between preoperative urine culture and pathogens causing PJI in this setting
- Step 3 – to co-design a guideline to reduce unnecessary preoperative testing for asymptomatic bacteriuria prior to elective arthroplasty
- Step 4 – to implement a sustainable guideline to reduce unnecessary preoperative testing for asymptomatic bacteriuria prior to elective arthroplasty

## 2 Methods

### 2.1 Setting

A 1200-bed central London teaching hospital, serving two local boroughs in addition to tertiary referrals, with over 900 arthroplasties performed annually.

### 2.2 Preoperative pathways

Patients undergoing arthroplasty are assessed by either the nurse-led preoperative assessment clinic supported by anaesthetic department (CPOAC) or by the geriatrician-led Perioperative Medicine for Older People undergoing Surgery (POPS) service. Patients are referred to POPS on the basis of multi-morbidity, geriatric syndromes, or complex decision making.

The remainder of the methodology will be discussed at each step of the programme of work.

#### 2.2.1 Step 1 – to describe current practice in preoperative urine testing prior to arthroplasty within a single centre

#### Methods

A retrospective observational case note review was undertaken in 100 patients preoperatively assessed by CPOAC and 100 patients preoperatively assessed by POPS prior to arthroplasty.

#### Results

Of the overall sample of 200 preoperative patients, 98 (49 %) had a MSU sent. Of these 98, 31 (32 %) reported at least one urinary symptom and 15 (15 %) tested positive for bacteriuria. Of the seven with a positive MSU result and symptoms, five (71 %) received antimicrobial treatment. Of eight patients with positive MSU results but no symptoms, six (75 %) were treated with antimicrobials.

#### 2.2.2 Step 2 – to examine the association between organisms isolated from preoperative urine culture and pathogens causing PJI within a single centre

#### Methods

A retrospective review of the hospital PJI database, 2012–2018, was undertaken. Data were collected on preoperative urine culture results, presence of urinary symptoms indicating UTI at time of culture and results of culture from joint.

#### Results

Sixty-one cases of PJI were identified between 2012 and 2018; during this time 5000 arthroplasties were performed at the centre. Eighteen of those patients (30 %) had a preoperative urine culture performed. One patient had the same pathogen (*E. coli*) in both the preoperative urine and joint culture. None of the patients had documentation of urinary symptoms at preoperative assessment when urine culture was sent.

#### 2.2.3 Step 3 – to co-design and implement a guideline to reduce unnecessary preoperative testing for asymptomatic bacteriuria prior to elective arthroplasty

#### Establishing a stakeholder group

All clinical stakeholders involved in the elective arthroplasty pathway were invited to participate in the co-design of the guideline. At the initial meeting, the results from steps 1 and 2 were presented by the POPS team and infectious diseases lead for PJI, at the orthopaedic clinical governance meeting. This meeting was attended by orthopaedic clinicians, preoperative assessment nurses, and the anaesthetic lead for preoperative assessment. The group achieved consensus in the need to effect change in preoperative testing for asymptomatic bacteriuria prior to elective arthroplasty. A smaller subgroup of stakeholders then co-designed a guideline based on Public Health England guidance (PHE, 2019): preoperative MSU testing in elective orthopaedic surgery with insertion of metalwork. According to usual hospital processes, the guideline was ratified by the antimicrobial stewardship committee.

#### Implementation phase

**Figure 2:**
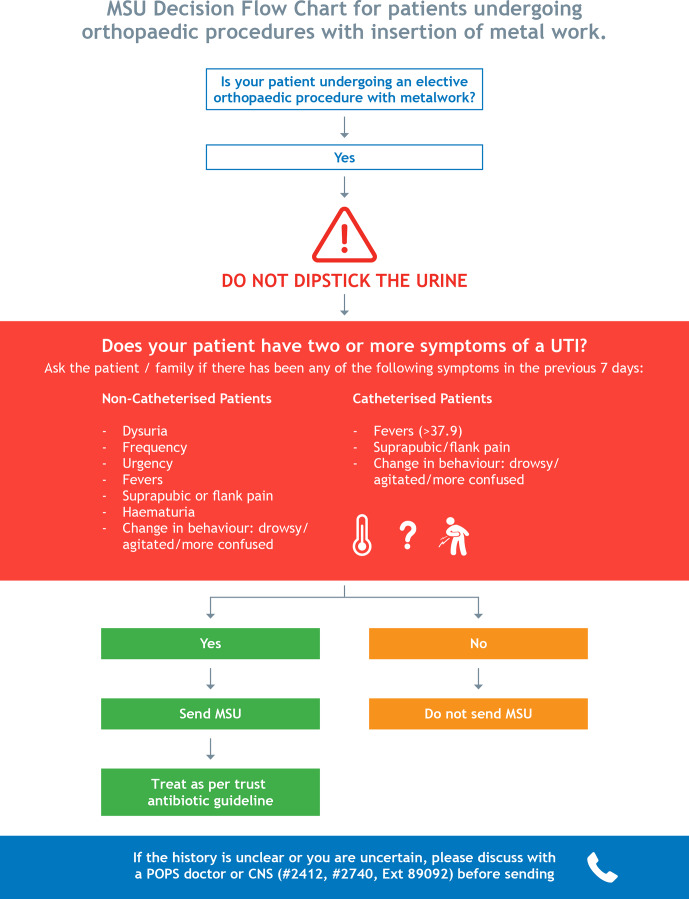
MSU decision flow chart guideline for preoperative MSU testing in elective orthopaedic surgery with insertion of metalwork.

The guideline was made available on the hospital intranet and was supported through a decision-aid tool co-produced by the stakeholder group and presented to the wider clinical team (Fig. 2: decision-aid tool to support implementation of the clinical guideline).

An implementation task force of junior doctors from the POPS team and senior nurses from the preoperative assessment team was established to support dissemination. Methods of dissemination included teaching sessions to explain the rationale behind the change to practice, visual aids to prompt awareness of the guideline and real-time feedback to all stakeholders. This feedback was provided through congratulatory poster emails, run charts of rates of adherence to new guidelines and update teaching sessions.

#### Data collection

Over a 3-month period, adherence to the guideline by the POPS and CPOAC teams was recorded. Patients undergoing elective arthroplasty assessed in the POPS clinic over a 1-month period were included (n=21). Due to the large number of patients seen by the CPOAC service, an alternative sampling frame was employed; patients undergoing elective arthroplasty seen by CPOAC on a single day each week for 4 weeks were included (n=25). Data were collected on preoperative urine culture and preoperative urinary symptoms suggestive of UTI. Cases of surgical site infection (SSI) obtained through the hospital surgical site infection database were recorded as a balancing measure.

#### Results

Following the implementation of the guideline, a total of two MSU samples were sent in the ensuing 3 months. In one case, the indication for the investigation was documented as symptoms of urinary urgency and new incontinence. The positive result was forwarded to the GP with instruction to treat based on antibiotic sensitivity. In the second case, the MSU was sent without clinical indication recorded. There were no surgical site infections related to orthopaedic implants entered into the hospital database for the 6-month period following implementation of the guideline.

#### 2.2.4 Step 4 – to evaluate the sustainability of a guideline to reduce unnecessary preoperative testing for asymptomatic bacteriuria prior to elective arthroplasty

At the end of Step 3, the implementation strategy was reviewed. The guideline remained on the hospital intranet, with open access for all clinicians. The decision support tool remained visible in all relevant clinical areas. Monthly emails were discontinued.

Six months post-intervention, adherence to the guideline was evaluated for 1 week.

No MSUs were sent in elective arthroplasty patients preoperatively assessed during this week (n=11 CPOAC, n=15 POPS). One inappropriate urine dipstick was performed – the results were not recorded and no antimicrobial treatment was prescribed.

## 3 Discussion

To our knowledge, this is the first article to describe the development and implementation of a guideline to reduce unnecessary preoperative testing for asymptomatic bacteriuria prior to elective arthroplasty. An initial review of practice at a single centre showed inconsistency in MSU testing and antimicrobial prescribing for UTI and ASB prior to elective arthroplasty. Retrospective evaluation did not show an association between organisms isolated from urine and joint aspirate in confirmed cases of PJI. These findings are in keeping with the literature that acknowledges clinical and financial benefits of avoiding unnecessary preoperative testing for ASB prior to elective arthroplasty (Lamb, 2016). Such non-adherence to national guidelines and consensus statements reflects the implementation gap between evidence and practice (David, 2000). In this study, a stepwise approach to design and implementation was undertaken. Early and comprehensive stakeholder engagement facilitated the rapid development of a guideline and decision support tool. A clear implementation strategy allowed widespread uptake over a short time period, with sustainability promoted through varied but targeted approaches to dissemination, including real-time feedback on adherence; this achieved the stated aim of reducing unnecessary testing for ASB prior to elective arthroplasty.

Challenging traditional healthcare practice can be difficult. In this study, application of stepwise methodology, with the inclusion of literature review (Sathanandan, 2019) and the use of implementation science methodology, provided a systematic approach to effecting change. This methodology is not dependent on complex technology or local infrastructure and is therefore easily translatable to other clinical settings, with potential cost savings across healthcare systems. Specifically, co-design involving all clinical stakeholders achieved buy-in, coherence, and cognitive participation from the outset. This engendered a culture of collective action and facilitated reflexive monitoring evidenced by sustainability of the intervention once the targeted prompt from the task force had been withdrawn. Such an approach has been described as an underpinning framework to develop, embed, and evaluate the implementation of complex interventions in a sustainable manner (May, 2009). Whilst the intervention in this study was not complex, the clinical pathway and number of stakeholders necessitated the use of methodology designed for implementation of multicomponent interventions (O'Cathain, 2019). Understanding of these issues and the use of appropriate methodology allowed longstanding practices to be disrupted.

Limitations to this study exist. Improvement science relies on regular, repeated measurement of change often presented through run charts. Whilst this approach was used, the dramatic reduction in MSU testing immediately following implementation and the low event rate made this technique less applicable. High levels of sustainability were achieved at 6 months, acknowledging the limitation of using a snapshot approach. Furthermore, the known limitations to retrospective chart review are acknowledged. For example, it was not possible to determine whether urinary symptoms were attributable to UTI or to other urinary tract pathology, such as prostatic enlargement and stress incontinence, common in an older patient population.

This work demonstrates that outdated clinical practice can be challenged using implementation science methodology. In this stepwise study, such an approach resulted in a reduction in unnecessary preoperative urine testing for ASB prior to elective arthroplasty. Widespread translation of this intervention has the potential to improve clinical care and achieve cost savings in an elective orthopaedic setting.

## Data Availability

Data is available upon request to the authors.
